# Influenza Virus-like Particle (VLP) Vaccines Expressing the SARS-CoV-2 S Glycoprotein, S1, or S2 Domains

**DOI:** 10.3390/vaccines9080920

**Published:** 2021-08-18

**Authors:** Ki-Back Chu, Hae-Ji Kang, Keon-Woong Yoon, Hae-Ahm Lee, Eun-Kyung Moon, Beom-Ku Han, Fu-Shi Quan

**Affiliations:** 1 Department of Biomedical Science, Graduate School, Kyung Hee University, Seoul 02447, Korea; kbchu@khu.ac.kr (K.-B.C.); haedi1202@naver.com (H.-J.K.); kgang92@gmail.com (K.-W.Y.); 2 Medical Research Center for Bioreaction to Reactive Oxygen Species and Biomedical Science Institute, School of Medicine, Graduate School, Kyung Hee University, Seoul 02447, Korea; hae.ahm.lee@gmail.com; 3 Department of Medical Zoology, School of Medicine, Kyung Hee University, Seoul 02447, Korea; ekmoon@khu.ac.kr; 4 Optipharm Inc., Cheongju 28158, Korea; bkhanhs@gmail.com

**Keywords:** COVID-19, SARS-CoV-2, virus-like particle, vaccine, antibody, neutralization

## Abstract

The ongoing severe acute respiratory syndrome coronavirus-2 (SARS-CoV-2) pandemic had brought disastrous consequences throughout the entire world. While several manufactured vaccines have been approved for emergency use, continuous efforts to generate novel vaccines are needed. In this study, we developed SARS-CoV-2 virus-like particles (VLPs) containing the full length of spike (S) glycoprotein (S full), S1, or S2 together with the influenza matrix protein 1 (M1) as a core protein. Successfully constructed VLPs expressing the S full, S1, and S2 via Sf9 cell transfections were confirmed and characterized by Western blot and transmission electron microscopy (TEM). VLP immunization in mice induced higher levels of spike protein-specific IgG and its subclasses compared to naïve control, with IgG2a being the most predominant subclass. S full and S1 immune sera elicited virus-neutralizing activities, but these were not strong enough to fully inhibit receptor–ligand binding of the SARS-CoV-2. Neutralizing activities were not observed from the S2 VLP immune sera. Overall, our findings revealed that S full or S1 containing VLPs can be developed into effective vaccines.

## 1. Introduction

Coronaviruses are single-stranded RNA viruses belonging to the order *Nidovirales*, family *Coronaviridae*, which can be subdivided into alphacoronavirus, betacoronavirus, and gammacoronavirus based on their antigenic and genetic characteristics [1]. While these viruses are frequently associated with zoonotic infections involving various avians and mammals, they are also capable of infecting humans which can be lethal as demonstrated by the severe acute respiratory syndrome (SARS) outbreak of 2003 [1,2]. In December of 2019, patients suffering from pneumonia of unknown etiology began to emerge in the city of Wuhan, China, whose cause was later attributed to the novel severe acute respiratory syndrome coronavirus 2 (SARS-CoV-2) classified under betacoronavirus [3,4]. Since then, the virus has spread to the entire world and global efforts to address this pandemic are currently ongoing. As of April 2021, 141 million confirmed cases of coronavirus disease–2019 (COVID-19) and 3 million deaths have been reported [5]. Clinical symptoms associated with SARS-CoV-2 infection include fever, dyspnea, and pneumonia, which could result in respiratory failure and death [6]. Yet, effective antiviral drugs remain unavailable and efficacious vaccines to control this outbreak are still under development [7]. Although several vaccines have been approved for emergency use by the United States Food and Drug Administration (FDA) [8], continued efforts to improve vaccine efficacy and safety are needed as indicated by newly emerging SARS-CoV-2 variants [9].

Given the current circumstances with the COVID-19 pandemic, virus-like particles (VLPs) are actively being investigated as a potential vaccine platform against the COVID-19. VLPs are highly immunogenic and considered superior compared to other traditional vaccines for several reasons. Because VLPs are completely devoid of viral genetic material required for replication, the vaccine is non-infectious and is safer than live-attenuated or whole-inactivated vaccines [10]. Notably, their small size allows rapid diffusion through the lymph nodes and facilitates antigen presentation for potent B and T cell inductions [10]. Currently, only one VLP-based SARS-CoV-2 vaccine (NVX-CoV2373, Novavax Inc., Gaithersburg, MD, American) is undergoing phase III clinical trial, and the interim results have been promising, with approximately 90% efficacy [11].

Several SARS-CoV-2 studies involving VLPs have been conducted and have reported interesting findings. Previously, Xu et al. [12] demonstrated that the roles of SARS-CoV-2 membrane protein and small envelope proteins are indispensable for VLP assembly. VLPs mimicking SARS-CoV-2 have been proposed as positive controls, and their role in clinical testing could remove some of the disparities in diagnosis [13]. VLPs expressing the spike (S) protein of SARS-CoV-2 have been demonstrated as a potential vaccine platform for the ongoing COVID-19 [14]. However, studies investigating the efficacy of the VLP-based vaccines against SARS-CoV-2 are limited. Recently, VLPs expressing the S protein receptor-binding domain (RBD) were reported to elicit potent neutralizing antibody responses in mice and pigs [15]. Similarly, substantial neutralizing antibody titers were observed from VLPs based on the Newcastle disease virus expressing the engineered pre-fusion stabilized S protein ectodomains [16]. While the results demonstrated by these two studies are promising, VLPs in these aforementioned studies were produced using mammalian cell lines. The major disadvantages of using mammalian cells are high production cost, low yield, and slower growth compared to other production methods [17]. A cheaper alternative to this method would be the insect cell-based baculovirus expression system, as these cells are relatively easier to handle and allow higher production yields in a shorter period of time compared to mammalian cells [18]. To date, studies investigating the SARS-CoV-2 neutralizing efficacy of insect cell-derived VLPs are non-existent. In the present study, VLPs expressing the S proteins (S full, S1, S2) of SARS-CoV-2 were generated using insect cells and antibody-mediated neutralization was evaluated.

## 2. Materials and Methods

### 2.1. Cells and Animals

*Spodoptera frugiperda* (Sf9) insect cells cultured in serum-free SF900 II medium (Invitrogen, Carlsbad, CA, USA) were used to generate recombinant baculovirus (rBV) and VLPs as described previously [19]. Twenty seven-week-old female BALB/c mice were purchased from NARA Biotech (Seoul, Korea) and subdivided into four groups (*n* = 5 per group). All animals were housed in an approved facility with day and night cycle, with easy access to food and water. All animal experimental procedures have been approved and conducted following the guidelines set out by Kyung Hee University IACUC (Permit number: KHSASP-20-666).

### 2.2. Codon Optimization, Gene Cloning, Recombinant Baculovirus, and VLP Production

Codon-optimized S full construct (3828 bp, GenBank accession number: QHD43416.1), S1 construct (2055 bp), and S2 construct (1764 bp) were synthesized from GenScript (Piscataway, NJ, USA). S1 construct was generated by fusing the transmembrane domain (TM) and cytoplasmic tail (CT) regions from the influenza virus hemagglutinin (HA) to the 3′ terminus of the S1 gene, and S2 construct was generated by fusing a signal peptide (SP) from honeybee melittin to the 5′ terminus of the S2 gene, respectively, as described [20]. Each of the codon-optimized genes was transformed into DH10Bac competent cells. Colony PCR and recombinant baculovirus productions were performed following the manufacturer’s instructions outlined in Bac-to-Bac Expression System (ThermoFisher, Waltham, MA, USA). Briefly, bacmid DNAs were transfected into Sf9 cells using Cellfectin II reagent for rBV production (Invitrogen, Carlsbad, CA, USA). VLPs were constructed by co-transfecting rBVs expressing each of the S full, S1, or S2 with influenza M1-expressing rBV using the method previously described [21]. After 3 days, transfected Sf9 cells were centrifuged at 6000 RPM for 30 min, 4 °C and supernatants were collected. Supernatants were ultracentrifuged at 30,000 RPM, 1 h, 4 °C and sedimented particles were resuspended in PBS overnight at 4 °C. The next day, particles were purified through a discontinuous sucrose gradient and ultracentrifuged at 30,000 RPM, 4 °C, 1 h. Faint bands, which correspond to the VLPs were carefully collected. VLPs were resuspended in PBS and ultracentrifuged at identical conditions to remove impurities. Pelleted VLPs were immersed in 100 μL of PBS and protein concentrations were measured using BCA assay kit (ThermoFisher, Waltham, MA, USA). The presence of baculoviruses budded particles in VLPs was determined by inoculating Sf9 cells with the purified and unpurified VLPs as previously described [22]. Briefly, Sf9 cells were seeded in 12-well culture plates and infected with rBV controls, unpurified VLPs (pre-sucrose), and purified VLPs (band 1, band 2). Post-sucrose was acquired from the uppermost supernatant layer. Band 1 and band 2 were collected from the two opaque bands that were located at the interfaces of 15%/30% and 30%/60% sucrose gradients, respectively. After collecting 1 mL of each fraction, protein assay was performed, and identical concentrations (5 μg) were inoculated into respective wells. Cells were monitored for 4 days to assess Sf9 cell infectivity. Images were acquired using a microscope (Leica Microsystems, Wetzlar, Germany).

### 2.3. Immunocytochemistry Using the Transfected rBVs

The polyclonal antibodies targeting the S RBD and the S2 domains were purchased from Sino Biological (Beijing, China) and these were used as primary antibodies for the immunocytochemistry. Immunocytochemistry using rBVs was performed as described elsewhere [23]. Briefly, Sf9 cells were transfected with the rBVs at MOI of 0.1, which were carefully collected 7 days after transfection and centrifuged at 1000 RPM for 3 min. For the washing steps, after aspirating the supernatant, pelleted cells were gently resuspended in PBS and centrifuged at 1000 RPM, 3 min, for a total of 3 times. Cells were blocked using 1% BSA in PBS with 0.1% Tween 20. Cells were washed three times with PBS and incubated with the primary antibodies targeting either the RBD (for S full and S1 rBV, 1:1000 dilution in PBS) or the S2 domain (for S2 rBV, 1:1000 dilution) for 1 h at 37 °C. Cells were washed thrice with PBS and incubated with anti-rabbit IgG secondary antibody (1:2000 dilution) conjugated with CFL-488 (Santa Cruz Biotechnology, Dallas, TX, USA) at 37 °C for 1 h. After final washing with PBS, cell suspensions were placed on a slide glass and mounted using the mounting medium containing 4′,6-diamidino-2-phenylindole (DAPI) (Vector Laboratories Inc., Burlingame, CA, USA). Images were acquired using a fluorescence microscope (Leica Microsystems, Wetzlar, Germany).

### 2.4. Characterization of VLPs

VLP constructs were characterized using Western blotting and transmission electron microscopy (TEM). Briefly, VLPs of various concentrations were separated via SDS-PAGE, and proteins were transferred to a nitrocellulose membrane. Membranes were blocked for 1 h at RT using 5% skim milk, and probed with the polyclonal antibodies purchased from Sino Biological (1:1000 dilution) overnight at 4 °C. The next day, membranes were washed three times with TBST and incubated with horseradish peroxidase (HRP)-conjugated secondary antibodies (1:2000 dilution) at RT for 1 h. After washing with TBST, bands were developed on X-ray film in the dark room using enhanced chemiluminescence (ECL). VLPs were observed under TEM. Briefly, samples were applied to glow-discharged carbon-coated copper grids and allowed to absorb for 2 min. After blotting off excess samples with Whatman paper, sample grids were stained with 2% uranyl acetate for 1 min. After removing excess reagents, results were recorded with Bio-High voltage EM system (JEM-1400 Plus at 120 kV and JEM-1000BEF at 1000 kV, JEOL Ltd., Tokyo, Japan) at Korea Basic Science Institute.

### 2.5. Animals, Immunization, and Sample Collection

Mice were divided into 4 groups (*n* = 5 per group): naïve (unimmunized), S full VLP, S1 VLP, and S2 VLP immunized. Mice were intramuscularly immunized through the quadriceps femoris with 100 μg of VLPs at 4-week intervals. Sera were collected 1 week after each immunization. All mice were sacrificed 4 weeks after the final immunization.

### 2.6. Antibody Responses against S1 and S2 Proteins of SARS-CoV-2

S1 and S2 antigens were purchased from Sino Biological (Beijing, China). Antigen-specific antibody responses were determined using ELISA, as described previously [24]. Briefly, 96-well plates were coated using identical concentrations of S1 and S2 antigens (both 1 μg/mL) combined in carbonate coating buffer overnight at 4 °C. S full VLPs were also coated at 1 μg/mL to assess successful immunization and antibody boosting effect. Wells were washed with PBST and blocked with 0.2% gelatin for 1 h, 37 °C. Diluted sera (1:100 dilution) of mice were added into wells and incubated for 1 h, 37 °C. After washing the wells three times with PBST, goat anti-mouse HRP-conjugated IgG, IgG1, IgG2a, and IgG2b secondary antibodies (1:2000 dilution; Southern Biotech, Birmingham, AL, USA) were inoculated into respective wells. Plates were incubated for 1 h, 37 °C and after final washing, 100 ul of o-phenylenediamine substrate (Sigma Aldrich, St. Louis, MO, USA) dissolved in citrate buffer with H_2_O_2_ were added into each well for colorimetric assay. Reactions were stopped with 2N H_2_SO_4_ and absorbance values at 450 nm were measured using EZ Read 400 microplate reader (Biochrom Ltd., Cambridge, UK).

### 2.7. Surrogate Virus Neutralization Assay (sVNT)

Surrogate virus neutralization assay, which allows assessment of virus inhibition without the BSL-3 facility requirement, was performed as described previously [25]. HRP-conjugated RBD was provided by Optipharm (Cheongju, Republic of Korea), which was performed using the HRP conjugation kit (Abcam, Cambridge, UK). SARS-CoV-2 human angiotensin-converting enzyme 2 (hACE2) receptor protein was purchased from GenScript (Piscataway, NJ, USA). After coating the plates with 100 ng of hACE2 using carbonate coating buffer in 96-well plates overnight at 4 °C, plates were washed with PBST and subsequently blocked with BD OptEIA assay diluent (BD Bioscience, San Diego, CA, USA) for 1 h at RT. Sera collected from mice 1 week after the final immunization were serially diluted in PBS and incubated at 56 °C for 30 min for complement inactivation. After heat inactivation, equal volumes of inactivated sera and HRP-conjugated RBD were mixed and inoculated into each well to be incubated for 1 h at 37 °C. Wells were washed with PBST and chromogenic reactions were developed using TMB substrate (BD Bioscience, San Diego, CA, USA). Reactions were stopped with 2N H_2_SO_4_ and absorbance values at 450 nm were measured. Percentage of inhibition was calculated as follows: Inhibition (%) = (1 − sample OD_450_/control OD_450_) × 100.

### 2.8. Statistical Analysis

Data sets are presented as mean ± SEM. Statistical analyses were performed using GraphPad Prism 5 software (San Diego, CA, USA). Statistical significance between groups were determined using one-way analysis of variance (ANOVA) with Tukey’s post hoc test. *p* values less than 0.05 were considered significant and are denoted using an asterisk.

## 3. Results

### 3.1. Transmembrane Protein Topology and Construct Schematic

**Figure 1:**
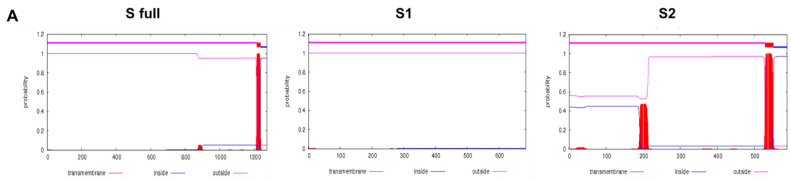

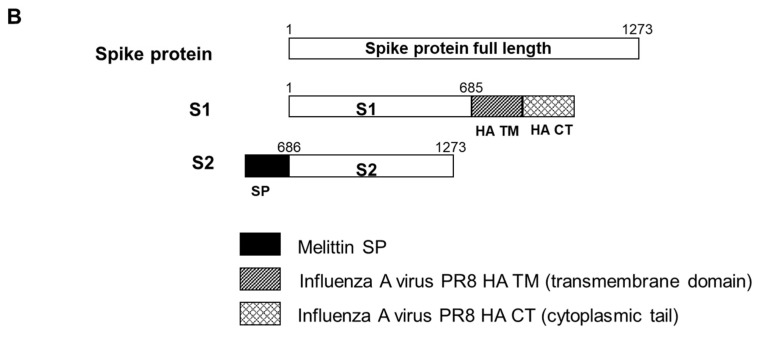
Transmembrane domain prediction and schematic diagram of the gene constructs. Original sequences of the S glycoprotein gene were analyzed by the TMHMM database and transmembrane protein topologies were predicted (**A**). Codon-optimized genes were constructed following the schematic illustrated above (**B**).

Transmembrane protein topologies were predicted using the TMHMM Server v.2.0 database. Original sequences of the full-length SARS-CoV-2 S glycoprotein, S1, and S2 domains were used for prediction (Figure 1A). Since the S1 portion of the protein did not contain any transmembrane domains, a transmembrane domain and cytoplasmic tail of the H1N1 influenza virus (A/Puerto Rico/8/34) was incorporated as described previously [20]. As the start codons were missing on the S2 domain, these were incorporated using the honey bee melittin signal peptide, which also enhances the expression of foreign protein expression in baculovirus-based insect cell lines [26]. A schematic diagram depicting the construction of S full, S1, and S2 codon-optimized genes is provided (Figure 1B). Codon-optimized HA TM and CT portions, along with the melittin SP have been used for the gene constructs. A full list of codon-optimized genes and the sequences have been provided in Table 1.

Codon-optimized sequences for S full gene, HA TM-CT, and melittin signal peptides were synthesized by GenScript. Nucleotide sequences for codon-optimization were acquired from the NCBI database (GenBank accession number: QHD43416.1, NC_002017.1, and NM_001011607.2).

### 3.2. Codon-Optimized Gene Cloning into pFastBac Vectors and Colony PCR

**Figure 2:**
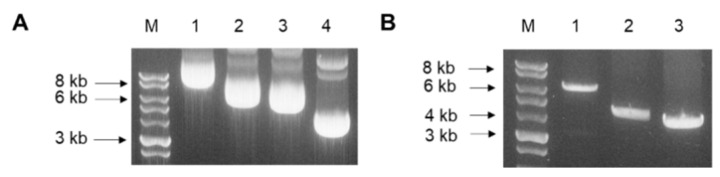
Successful cloning of S protein inserts. Codon-optimized genes were successful cloned into pFastBac vector (**A**). Vectors containing each insert were transformed into DH10Bac competent cells and bacmids were analyzed via colony PCR (**B**). Lane identifications for both panels are as follows: M, marker; 1, S full; 2, S1; 3, S2; 4, pFastBac vector.

Codon-optimized S full, S1, and S2 genes were transformed into pFastBac vectors. Successful cloning of the genes was confirmed through gel electrophoresis. Total gene lengths for S full, S1, S2, and pFastBac vectors were 8.7 kbp, 6.7 kbp, 6.4 kbp, and 4.8 kbp, respectively (Figure 2A). Vectors containing the spike protein inserts were subsequently transformed into DH10Bac competent cells. To confirm successful transformation of the plasmids, recombinant bacmid DNA was analyzed by colony PCR. Since bacmid transposed with the pFastBac vector is roughly 2.3 kbp, the spike protein inserts were correctly transformed, as indicated by the size differences (Figure 2B).

### 3.3. Construction of Spike Protein-Expressing Recombinant Baculoviruses

**Figure 3:**
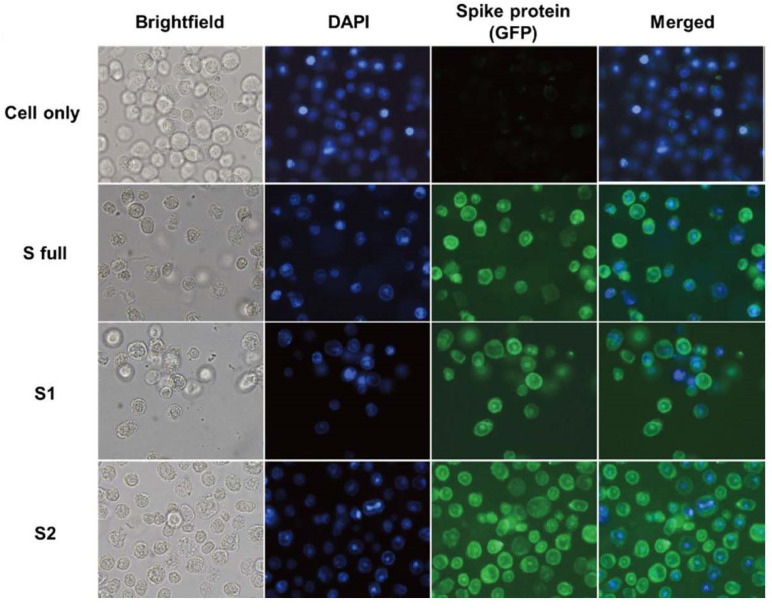
Confirming recombinant baculovirus construction. Polyclonal antibodies were raised against the S RBD and S2 antigens. The two antibodies were used to detect successful transfection of bacmid DNAs into Sf9 cells via immunocytochemistry. The S RBD antibodies were used to detect protein expression for the S full and S1 rBVs, whereas S2 antibodies were specifically used for the S2 rBV.

Polyclonal antibodies targeting the S protein RBD and the S2 were assessed for their binding capability. Immunocytochemistry was performed using these polyclonal antibodies and the recombinant baculoviruses. While Sf9 cell nuclei were stained with DAPI as expected, fluorescence on the cell surfaces was not observed even when incubated with the polyclonal antibodies. Since both S full and S1 proteins contain the RBDs, the polyclonal antibody raised against the S RBD was used to confirm successful rBV expression. Polyclonal S2 antibody also reacted with the S2 protein-expressing rBVs (Figure 3).

### 3.4. Characterization of VLPs by Western blot and TEM

**Figure 4:**
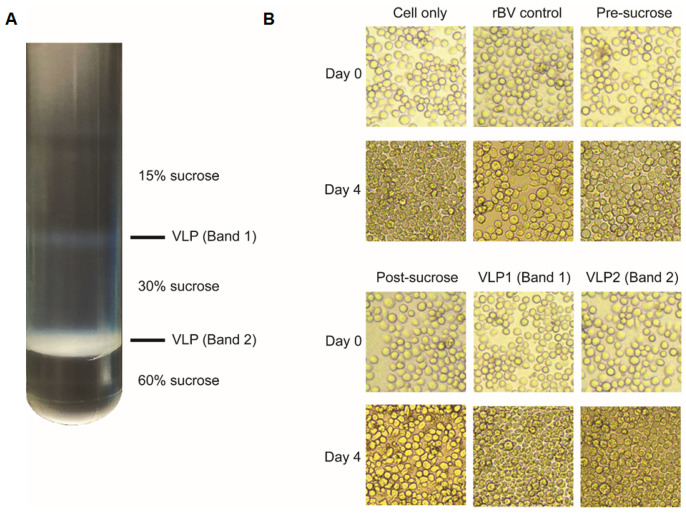
Confirming baculovirus removal after sucrose gradient purification. S full VLPs were used as a representative to confirm baculovirus removal from the VLPs described in this study. Sf9 cells were inoculated with fractions of S full VLPs acquired post-sucrose purification, rBV control, and VLPs before purification (pre-sucrose). Non-inoculated Sf9 cells were used as a negative control. Purified VLPs were labeled as VLP1 (band 1) and VLP2 (band 2) (**A**). Sf9 cells were monitored daily for 4 days to assess baculovirus cell infectivity under the microscope (**B**). All images were acquired at 100× magnification.

As illustrated, two distinct opaque bands corresponding to the VLP1 and VLP2 were formed at the interfaces between the sucrose layers (Figure 4A). Pre-sucrose, post-sucrose, VLP1, and VLP2 were collected and used to infect Sf9 cells. The acquired images (Figure 4B) showed that VLP1- and 2-treated cells continued to proliferate well at day 4, similar to uninfected cells, indicating that the majority of the baculovirus particles had been removed from VLP1 and VLP2. However, rBV control-, pre-sucrose-, and post-sucrose-treated Sf9 cells underwent cell death, enlargement, and did not proliferate as the other groups did, indicating that baculoviruses were present.

**Figure 5:**
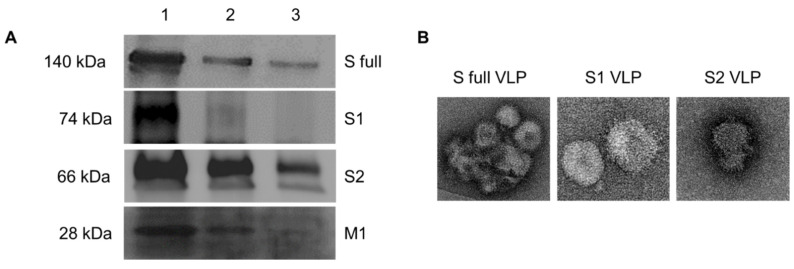
VLPs were characterized by Western blot and TEM. Expressions of the S full, S1, S2, and M1 proteins in the VLPs were evaluated by Western blot. Lanes 1, 2, and 3 correspond to protein loading concentrations of 40 μg, 20 μg, and 10 μg, each respectively (**A**). VLP images were acquired under TEM (**B**).

To confirm proper construction of the VLPs, Western blotting and TEM analyses were conducted. Consistent with the rBV immunocytochemistry data, the polyclonal antibodies used in the study successfully interacted with the VLPs expressing S full, S1, or S2 proteins. When VLPs transferred onto the nitrocellulose membranes were probed with the polyclonal S RBD antibody, S full and S1 VLPs around 140 kDa and 74 kDa were detected which corresponds to their molecular weight. Similarly, probing S2 VLPs with the S2 polyclonal antibody resulted in bands developing around 66 kDa. Probing the VLPs with the M1 monoclonal antibody revealed that the recombinant baculoviruses expressing the spike proteins were successfully displayed on the M1 protein (Figure 5A). TEM analysis revealed successful display of S full, S1, and S2 proteins on the surface of VLPs (Figure 5B).

### 3.5. VLP Immunization Induced Antibody Response in Sera

**Figure 6:**
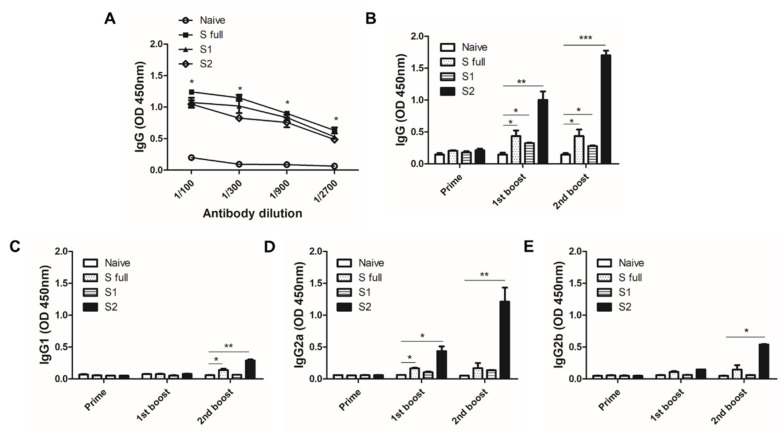
Antigen-specific antibody responses induced by VLP immunization. Sera of mice were collected 1 week after each immunization and were used to assess the antigen-specific antibody responses via ELISA. VLP immunization and boosting effect of VLP immunization was confirmed by reacting the sera against S full VLP (**A**). All of the sera collected over the course of the animal studies were used to observe changes in IgG (**B**), IgG1 (**C**), IgG2a (**D**), IgG2b (**E**) responses against S1 and S2 antigens. Data are expressed as mean ± SEM (* *p* < 0.05, ** *p* < 0.01, *** *p* < 0.001).

To confirm the immunogenicity of the VLPs, 100 μg of the VLPs were intramuscularly administered in mice. At 1 week after the second immunization, blood of mice was collected and ELISA was performed to confirm successful immunization and boosting effect. All of the sera collected from each immunized group reacted against the S full VLPs and induced potent antibody response, whereas naïve sera failed to elicit significant antibody responses (Figure 6A). To check whether the antibody responses induced by VLPs could detect the S1 and S2 subunits of the S protein, S1 and S2 antigens were coated and ELISA was performed. Surprisingly, 1 week after the 1st boost, the strongest IgG antibody response induction was observed from S2 VLP-immunized mice. Compared to naïve control, significant antibody response inductions were also observed from S full and S1 VLP immune sera. A similar trend was observed from sera following the 2nd boost immunization, with the highest induction being observed from S2 VLP immune sera. However, enhanced antibody inductions after the 2nd boost for S full and S1 VLP immune sera were not detected (Figure 6B). IgG1 antibody responses were induced to a significant level by S full and S2 VLP immunizations, but noticeable changes were not observed until final immunization (Figure 6C). S protein-specific IgG2a responses were similar to those of IgG. Potent antibody responses were demonstrated from sera of mice immunized with the S2 VLP. While S full VLP induced elicited significantly enhanced IgG2a response following the 1st boost, subsequent immunization had negligible impact on its induction (Figure 6D). IgG2b subclass response was more or less similar to IgG1 response, with significant induction only observed from S2 VLPs after the 2nd boost immunization (Figure 6E).

### 3.6. Effective Inhibition Requires Antibodies Raised against the S1 Domain

**Figure 7:**
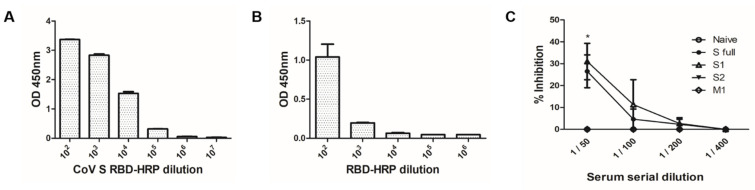
Surrogate virus neutralization requires the presence of S1 domain. Confirmation of HRP conjugation to S RBD (**A**) and successful RBD-HRP interaction with the hACE2 (**B**) was assessed through ELISA. Surrogate virus neutralization assay was conducted using serially diluted sera of immunized mice after heat-inactivation (**C**). Data are expressed as mean ± SEM (* *p* < 0.05 vs. naïve control).

To confirm whether the antibody responses induced by VLPs could inhibit the viruses, sVNT was conducted. Successful conjugation of HRP to the S RBD was assessed through ELISA. Upon reaction with the TMB substrate, a potent colorimetric response was immediately developed thereby indicating that HRP conjugation to the RBD has occurred (Figure 7A). To check whether the RBD-HRP is capable of binding to the hACE2 receptor, ELISA was performed. Serially diluted RBD-HRP in PBS was reacted with 100 ng of hACE2 and the strongest response was observed from 10^2^ dilution (Figure 7B). Based on these experimental conditions, 100 ng of hACE2 and 10^2^ diluted RBD-HRP mixture were selected for sVNT assay. Sera of naïve mice did not confer any neutralizing activity as expected. Interestingly, despite the potent antibody response induced by S2 VLPs, it failed to inhibit RBD binding to the hACE2 receptor. On the contrary, VLPs possessing the S1 domain conferred partial inhibition of RBD-hACE2 binding (Figure 7C). The strongest RBD-inhibiting activity was observed at 1:50 serum dilution for both of the S full and S1 mice sera, which drastically diminished following further dilutions.

## 4. Discussion

The COVID-19 pandemic continues to wreak havoc across the globe and vaccines are direly needed. In this study, we generated VLP vaccines expressing the S full, S1, or S2 glycoprotein of the SARS-CoV-2 using the insect cell-based baculovirus expression system. After characterization, the immunogenicities were evaluated in mice. Our results illustrate that baculovirus-expressed VLPs displaying the S or S1 proteins are capable of eliciting antibody responses contributing to viral inhibition as demonstrated through sVNT assay. With multiple reports of emerging SARS-CoV-2 variants that include mutations in the RBD [27,28], raising antibodies against a more conserved region that contributes to neutralization is desired. In the case of influenza viruses, vaccines based on the conserved HA stalk domain provided protection against challenge infection in mice and ferrets [29,30,31]. Additionally, influenza viruses possessing mutated stalk domain failed to evade the host immune response induced by stalk-based vaccines in mice [32]. Using this rationale, we anticipated that antibodies raised against the stalk domain S2 of SARS-CoV-2 could be protective and may even be used to confer resistance against the newly emerging variants. Our findings revealed that potent antibody responses against the S2 domain were induced over the course of three immunizations. Vaccination with the S full and S1 VLPs also induced significant increases in the antigen-specific IgG and IgG2a responses, though the extent to which these were induced paled in comparison to that of S2 VLPs.

Our ELISA results demonstrated that the Th1-associated IgG2a was induced to a significantly greater extent than the Th2-associated IgG1, particularly from S2 VLPs, thus implying potential Th1-bias in the elicited immune response. Similar to the present findings, orally administering a yeast-based vaccine expressing the spike glycoprotein of SARS-CoV-2 elicited a mixed Th1/Th2 immune response with a Th1 bias in BALB/c mice [33]. The presence of functional T cell responses, particularly CD4^+^ and CD8^+^ T cells, is important for SARS-CoV-2 patients. Reportedly, T cell exhaustion and extremely low levels of peripheral T cells were common features observed in COVID-19 patients [34]. Given the nature of VLPs, which are capable of inducing both humoral and cellular immune responses [35], robust expansion of immune cells associated with cellular immunity could be expected from the VLP vaccines presented here.

Antibodies raised against internal structural proteins such as M1 have been reported to be incapable of neutralizing the influenza virus, which stems from their relative inaccessibility of these proteins compared to the HA or the NA surface antigens [36]. As such, M1 VLP sera failed to inhibit RBD-hACE2 binding as was the case for the naïve sera. IgG antibody responses to the SARS-CoV-2 RBD have previously been reported to be associated with potent neutralizing activity [37]. While potent IgG and IgG2a antibody responses were induced by S2 VLP immunization in our study, which exceeded those elicited by S full and S1 VLPs, S2 VLP immune sera failed to inhibit RBD binding with the hACE2. This was as expected, since the RBD is absent on the S2 stalk domain [38]. Rather, partial RBD-hACE2 binding inhibition was observed from sera of mice immunized with the VLPs possessing the S1 domain. This is consistent with the current literature reporting that neutralizing antibody responses were directed towards the RBD-containing S1 domain of the S glycoprotein [39,40]. The first sVNT described by Tan et al. [25] was conducted using the sera of convalescing COVID-19 patients with a cutoff value of 30%, and effectively detected the presence of SARS-CoV-2 virus-neutralizing antibodies. However, our VLP vaccine-induced antibodies partially inhibited RBD-hACE2 binding which barely exceeded the aforementioned cutoff value. While this can be interpreted as a lack of neutralizing antibody titers in sera, it is important to note that virus neutralization may not always be dependent on the presence of neutralizing antibodies. In support of our finding, a clinical study involving convalescing SARS-CoV-2 patients reported that neutralizing antibody responses were quite varied. Specifically, multiple patients have been reported to possess neutralizing antibody titers far below the detection limit, but these patients still managed to recover from the disease and the duration of the symptoms were more or less similar to those with detectable neutralizing antibody titers [41].

One possible explanation for the phenomenon described above is the presence of non-neutralizing antibodies. While sera acquired from SARS-CoV-2-invected patients and several monoclonal antibodies have been demonstrated to neutralize the virus, this was not necessarily always the case. A noticeable level of non-neutralizing antibodies has been detected in the sera of infected patients, and even some monoclonal antibodies targeting the S protein have been found to be non-neutralizing from multiple neutralization assays [42]. Additionally, clinical findings have revealed that patients exposed to other types of human coronavirus prior to the COVID-19 pandemic possessed antibodies that could cross-react with the S proteins of currently circulating SARS-CoV-2. These antibody levels, albeit failing to contribute to protection via virus neutralization, were reported to be elevated following SARS-CoV-2 infection [43]. Moreover, sera collected from COVID-19 patients were capable of reacting with the OC43 betacoronavirus and predominantly targeted the S2 domain, rather than the S1 domain of the OC43 S protein [43]. Non-neutralizing antibodies could also be involved in the poor neutralization results demonstrated by the antibodies raised against the S1 domain. When neutralizing and non-neutralizing antibodies of SARS-CoV were combined in a virus neutralization assay, non-neutralizing antibodies were reported to interfere with the virus inhibition process [44]. It is important to note that the antibody response directed at the S2 domain demonstrated in our study could be neutralizing or non-neutralizing. Although discerning the nature of these S2-specific antibodies was beyond the scope of this study, further studies should be conducted to unravel their role in COVID-19 protection.

While non-neutralizing antibodies are perceived as a detriment to protection against SARS-CoV-2, this may not necessarily be the case, as demonstrated in the case of other pulmonary viruses. Non-neutralizing antibodies confer protection via antibody-mediated effector functions including the antibody-dependent cellular cytotoxicity [45,46]. A study involving respiratory syncytial virus reported that non-neutralizing antibodies raised against the attachment glycoprotein failed to neutralize the virus in vitro but was positively correlated with protection [47]. Recently, the potential role of non-neutralizing antibodies in protection against SARS-CoV-2 was suggested. Noticeable improvements to virus neutralization were demonstrated by bispecific antibodies engineered to express both neutralizing and non-neutralizing epitopes of the S RBD compared to the control groups [48]. As such, unraveling the function of these non-neutralizing antibodies and their involvement in protection against SARS-CoV-2 could be beneficial. Additionally, since multi-antigenic vaccines have been reported to confer broader and enhanced protection compared to single antigen vaccines against various diseases [49,50,51], combining S1 and S2 epitopes may also be a potential vaccination strategy. Further studies assessing the VLP vaccine efficacy using animal models such as ferrets and Syrian golden hamsters would be ideal for in vivo studies since these animals are susceptible to SARS-CoV-2 with clinical symptoms and pathogenicity being not too dissimilar to those found in humans [52,53].

## 5. Conclusions

In summary, our study revealed that immunizing mice with the VLPs expressing the S glycoprotein induces S protein-specific antibody responses, with those elicited by the S2 VLP being particularly potent. While future studies investigating the efficacy of VLP vaccines expressing a diverse array of SARS-CoV-2 neutralizing epitopes would be interesting and could possibly become a promising COVID-19 therapeutic option, additional studies are warranted to elucidate the role of non-neutralizing antibodies in protection against the ongoing pandemic.

## Figures and Tables

**Table 1:** Codon-optimized sequences of the SARS-CoV-2 S glycoprotein, influenza HA TM-CT, and melittin signal peptide.

Optimized 1	ATGTTCGTTTTCCTCGTGCTCCTCCCCCTCGTTTCCTCCCAATGCGTCAACCTCACTACC
Original 1	ATGTTTGTTTTTCTTGTTTTATTGCCACTAGTCTCTAGTCAGTGTGTTAATCTTACAACC
Optimized 61	CGTACCCAGCTCCCACCAGCCTACACCAACAGCTTCACTCGCGGTGTGTACTACCCCGAC
Original 61	AGAACTCAATTACCCCCTGCATACACTAATTCTTTCACACGTGGTGTTTATTACCCTGAC
Optimized 121	AAGGTCTTCCGTTCCAGCGTGCTGCACAGCACTCAGGACCTGTTCCTGCCATTCTTCTCT
Original 121	AAAGTTTTCAGATCCTCAGTTTTACATTCAACTCAGGACTTGTTCTTACCTTTCTTTTCC
Optimized 181	AACGTGACCTGGTTCCACGCCATCCACGTGAGCGGCACCAACGGCACTAAGCGCTTCGAC
Original 181	AATGTTACTTGGTTCCATGCTATACATGTCTCTGGGACCAATGGTACTAAGAGGTTTGAT
Optimized 241	AACCCTGTGCTGCCCTTCAACGACGGTGTCTACTTCGCTTCCACCGAGAAGTCTAACATC
Original 241	AACCCTGTCCTACCATTTAATGATGGTGTTTATTTTGCTTCCACTGAGAAGTCTAACATA
Optimized 301	ATCCGTGGATGGATCTTCGGCACCACTCTGGACTCAAAGACTCAGTCCCTGCTGATCGTC
Original 301	ATAAGAGGCTGGATTTTTGGTACTACTTTAGATTCGAAGACCCAGTCCCTACTTATTGTT
Optimized 361	AACAACGCCACCAACGTGGTCATCAAGGTGTGCGAGTTCCAGTTCTGCAACGACCCTTTC
Original 361	AATAACGCTACTAATGTTGTTATTAAAGTCTGTGAATTTCAATTTTGTAATGATCCATTT
Optimized 421	CTGGGCGTCTACTACCACAAGAACAACAAGAGCTGGATGGAGTCTGAGTTCCGCGTCTAC
Original 421	TTGGGTGTTTATTACCACAAAAACAACAAAAGTTGGATGGAAAGTGAGTTCAGAGTTTAT
Optimized 481	TCTTCAGCTAACAACTGCACTTTCGAGTACGTGAGCCAGCCCTTCCTGATGGACCTGGAA
Original 481	TCTAGTGCGAATAATTGCACTTTTGAATATGTCTCTCAGCCTTTTCTTATGGACCTTGAA
Optimized 541	GGAAAGCAGGGTAACTTCAAGAACCTGAGGGAGTTCGTGTTCAAGAACATCGACGGATAC
Original 541	GGAAAACAGGGTAATTTCAAAAATCTTAGGGAATTTGTGTTTAAGAATATTGATGGTTAT
Optimized 601	TTCAAGATTTACTCAAAGCACACCCCTATCAACCTGGTGCGCGACCTGCCACAGGGTTTC
Original 601	TTTAAAATATATTCTAAGCACACGCCTATTAATTTAGTGCGTGATCTCCCTCAGGGTTTT
Optimized 661	TCCGCTCTGGAGCCTCTGGTGGACCTGCCCATCGGCATCAACATCACCCGCTTCCAGACT
Original 661	TCGGCTTTAGAACCATTGGTAGATTTGCCAATAGGTATTAACATCACTAGGTTTCAAACT
Optimized 721	CTGCTGGCTCTGCACCGTTCCTACCTGACTCCTGGCGACTCCAGCTCTGGATGGACCGCC
Original 721	TTACTTGCTTTACATAGAAGTTATTTGACTCCTGGTGATTCTTCTTCAGGTTGGACAGCT
Optimized 781	GGAGCTGCCGCTTACTACGTGGGTTACCTGCAACCCAGGACCTTCCTGCTGAAGTACAAC
Original 781	GGTGCTGCAGCTTATTATGTGGGTTATCTTCAACCTAGGACTTTTCTATTAAAATATAAT
Optimized 841	GAAAACGGAACCATCACAGACGCTGTGGACTGCGCTCTGGACCCCCTGAGCGAAACCAAG
Original 841	GAAAATGGAACCATTACAGATGCTGTAGACTGTGCACTTGACCCTCTCTCAGAAACAAAG
Optimized 901	TGCACTCTGAAGTCTTTCACCGTGGAGAAGGGCATCTACCAGACTAGCAACTTCAGGGTG
Original 901	TGTACGTTGAAATCCTTCACTGTAGAAAAAGGAATCTATCAAACTTCTAACTTTAGAGTC
Optimized 961	CAGCCAACCGAATCTATCGTCAGATTCCCCAACATCACTAACCTGTGCCCATTCGGAGAG
Original 961	CAACCAACAGAATCTATTGTTAGATTTCCTAATATTACAAACTTGTGCCCTTTTGGTGAA
Optimized 1021	GTCTTCAACGCCACCAGATTCGCTTCCGTGTACGCCTGGAACAGGAAGAGAATCAGCAAC
Original 1021	GTTTTTAACGCCACCAGATTTGCATCTGTTTATGCTTGGAACAGGAAGAGAATCAGCAAC
Optimized 1081	TGCGTCGCTGACTACTCTGTGCTGTACAACAGCGCCTCTTTCTCAACCTTCAAGTGCTAC
Original 1081	TGTGTTGCTGATTATTCTGTCCTATATAATTCCGCATCATTTTCCACTTTTAAGTGTTAT
Optimized 1141	GGCGTGAGCCCTACTAAGCTGAACGACCTGTGCTTCACCAACGTCTACGCCGACTCTTTC
Original 1141	GGAGTGTCTCCTACTAAATTAAATGATCTCTGCTTTACTAATGTCTATGCAGATTCATTT
Optimized 1201	GTGATCAGGGGAGACGAGGTCAGACAGATCGCTCCCGGCCAGACTGGAAAGATCGCCGAC
Original 1201	GGAGTGTCTCCTACTAAATTAAATGATCTCTGCTTTACTAATGTCTATGCAGATTCATTT
Optimized 1261	TACAACTACAAGCTGCCAGACGACTTCACCGGCTGCGTCATCGCTTGGAACTCAAACAAC
Original 1261	TATAATTATAAATTACCAGATGATTTTACAGGCTGCGTTATAGCTTGGAATTCTAACAAT
Optimized 1321	CTGGACTCCAAAGTGGGTGGCAACTACAACTACCTGTACCGCCTGTTCCGTAAGAGCAAC
Original 1321	CTTGATTCTAAGGTTGGTGGTAATTATAATTACCTGTATAGATTGTTTAGGAAGTCTAAT
Optimized 1381	CTGAAGCCTTTCGAGAGGGACATCTCAACTGAAATCTACCAGGCTGGTTCCACCCCCTGC
Original 1381	CTCAAACCTTTTGAGAGAGATATTTCAACTGAAATCTATCAGGCCGGTAGCACACCTTGT
Optimized 1441	AACGGTGTCGAGGGCTTCAACTGCTACTTCCCACTGCAATCTTACGGTTTCCAGCCTACT
Original 1441	AATGGTGTTGAAGGTTTTAATTGTTACTTTCCTTTACAATCATATGGTTTCCAACCCACT
Optimized 1501	AACGGTGTGGGCTACCAGCCCTACAGAGTGGTCGTGCTGTCATTCGAACTGCTGCACGCC
Original 1501	AATGGTGTTGGTTACCAACCATACAGAGTAGTAGTACTTTCTTTTGAACTTCTACATGCA
Optimized 1561	CCAGCTACTGTGTGCGGTCCTAAGAAGTCCACCAACCTGGTCAAGAACAAGTGCGTGAAC
Original 1561	CCAGCAACTGTTTGTGGACCTAAAAAGTCTACTAATTTGGTTAAAAACAAATGTGTCAAT
Optimized 1621	TTCAACTTCAACGGCCTGACCGGAACTGGTGTCCTGACCGAGTCAAACAAGAAGTTCCTG
Original 1621	TTCAACTTCAATGGTTTAACAGGCACAGGTGTTCTTACTGAGTCTAACAAAAAGTTTCTG
Optimized 1681	CCATTCCAGCAGTTCGGAAGGGACATCGCTGACACCACTGACGCTGTGCGCGACCCTCAG
Original 1681	CCTTTCCAACAATTTGGCAGAGACATTGCTGACACTACTGATGCTGTCCGTGATCCACAG
Optimized 1741	ACCCTGGAAATCCTGGACATCACTCCTTGCAGCTTCGGAGGTGTCTCTGTGATCACCCCT
Original 1741	ACACTTGAGATTCTTGACATTACACCATGTTCTTTTGGTGGTGTCAGTGTTATAACACCA
Optimized 1801	GGCACCAACACTTCCAACCAGGTCGCTGTGCTGTACCAGGACGTCAACTGCACCGAGGTG
Original 1801	GGAACAAATACTTCTAACCAGGTTGCTGTTCTTTATCAGGATGTTAACTGCACAGAAGTC
Optimized 1861	CCTGTGGCTATCCACGCTGACCAGCTGACCCCAACTTGGCGCGTGTACTCCACCGGCTCC
Original 1861	CCTGTTGCTATTCATGCAGATCAACTTACTCCTACTTGGCGTGTTTATTCTACAGGTTCT
Optimized 1921	AACGTCTTCCAGACTCGTGCTGGTTGCCTGATCGGCGCCGAGCACGTGAACAACTCATAC
Original 1921	AATGTTTTTCAAACACGTGCAGGCTGTTTAATAGGGGCTGAACATGTCAACAACTCATAT
Optimized 1981	GAATGCGACATCCCAATCGGCGCTGGAATCTGCGCCTCCTACCAGACCCAGACTAACTCA
Original 1981	GAGTGTGACATACCCATTGGTGCAGGTATATGCGCTAGTTATCAGACTCAGACTAATTCT
Optimized 2041	CCTCGCCGTGCTCGCTCCGTCGCCTCCCAGAGCATCATCGCTTACACCATGAGCCTGGGC
Original 2041	CCTCGGCGGGCACGTAGTGTAGCTAGTCAATCCATCATTGCCTACACTATGTCACTTGGT
Optimized 2101	GCTGAAAACTCTGTGGCCTACTCCAACAACAGCATCGCCATCCCAACCAACTTCACTATC
Original 2101	GCAGAAAATTCAGTTGCTTACTCTAATAACTCTATTGCCATACCCACAAATTTTACTATT
Optimized 2161	TCAGTGACCACTGAGATCCTGCCTGTCTCAATGACCAAGACTTCCGTGGACTGCACTATG
Original 2161	AGTGTTACCACAGAAATTCTACCAGTGTCTATGACCAAGACATCAGTAGATTGTACAATG
Optimized 2221	TACATCTGCGGAGACTCAACCGAATGCTCCAACCTGCTGCTGCAATACGGCTCCTTCTGC
Original 2221	TACATTTGTGGTGATTCAACTGAATGCAGCAATCTTTTGTTGCAATATGGCAGTTTTTGT
Optimized 2281	ACCCAGCTGAACCGTGCTCTGACTGGAATCGCCGTGGAGCAGGACAAGAACACTCAGGAA
Original 2281	ACACAATTAAACCGTGCTTTAACTGGAATAGCTGTTGAACAAGACAAAAACACCCAAGAA
Optimized 2341	GTCTTCGCTCAGGTGAAGCAAATCTACAAGACCCCTCCCATCAAGGACTTCGGCGGATTC
Original 2341	GTTTTTGCACAAGTCAAACAAATTTACAAAACACCACCAATTAAAGATTTTGGTGGTTTT
Optimized 2401	AACTTCTCCCAGATCCTGCCCGACCCATCTAAGCCTTCAAAGCGCTCCTTCATCGAGGAC
Original 2401	AATTTTTCACAAATATTACCAGATCCATCAAAACCAAGCAAGAGGTCATTTATTGAAGAT
Optimized 2461	CTGCTGTTCAACAAGGTCACCCTGGCCGACGCTGGATTCATCAAGCAGTACGGAGACTGC
Original 2461	CTACTTTTCAACAAAGTGACACTTGCAGATGCTGGCTTCATCAAACAATATGGTGATTGC
Optimized 2521	CTGGGTGACATCGCCGCTCGTGACCTGATCTGCGCTCAGAAGTTCAACGGTCTGACCGTG
Original 2521	CTTGGTGATATTGCTGCTAGAGACCTCATTTGTGCACAAAAGTTTAACGGCCTTACTGTT
Optimized 2581	CTGCCACCTCTGCTGACTGACGAAATGATCGCCCAGTACACTTCAGCCCTGCTGGCTGGA
Original 2581	TTGCCACCTTTGCTCACAGATGAAATGATTGCTCAATACACTTCTGCACTGTTAGCGGGT
Optimized 2641	ACCATCACTTCCGGTTGGACCTTCGGTGCTGGTGCTGCTCTGCAAATCCCCTTCGCTATG
Original 2641	ACAATCACTTCTGGTTGGACCTTTGGTGCAGGTGCTGCATTACAAATACCATTTGCTATG
Optimized 2701	CAGATGGCCTACAGGTTCAACGGAATCGGTGTCACCCAGAACGTGCTGTACGAGAACCAG
Original 2701	
Optimized 2761	CAAATGGCTTATAGGTTTAATGGTATTGGAGTTACACAGAATGTTCTCTATGAGAACCAA
Original 2761	AAGCTGATCGCTAACCAGTTCAACAGCGCCATCGGAAAGATCCAGGACTCACTGTCATCC
Optimized 2821	AAATTGATTGCCAACCAATTTAATAGTGCTATTGGCAAAATTCAAGACTCACTTTCTTCC
Original 2821	ACTGCCTCCGCTCTGGGCAAGCTGCAAGACGTCGTGAACCAGAACGCCCAGGCTCTGAAC
Optimized 2881	ACAGCAAGTGCACTTGGAAAACTTCAAGATGTGGTCAACCAAAATGCACAAGCTTTAAAC
Original 2881	ACCCTGGTCAAGCAGCTGTCCTCCAACTTCGGTGCTATCTCATCCGTGCTGAACGACATC
Optimized 2941	ACGCTTGTTAAACAACTTAGCTCCAATTTTGGTGCAATTTCAAGTGTTTTAAATGATATC
Original 2941	CTGTCTCGCCTGGACAAGGTCGAGGCCGAAGTGCAGATCGACCGCCTGATCACCGGCCGC
Optimized 3001	CTTTCACGTCTTGACAAAGTTGAGGCTGAAGTGCAAATTGATAGGTTGATCACAGGCAGA
Original 3001	CTGCAATCCCTGCAAACCTACGTGACTCAGCAGCTGATCAGGGCCGCTGAAATCAGAGCC
Optimized 3061	CTTCAAAGTTTGCAGACATATGTGACTCAACAATTAATTAGAGCTGCAGAAATCAGAGCT
Original 3061	TCTGCTAACCTGGCCGCTACCAAGATGTCAGAGTGCGTCCTGGGTCAGTCCAAGCGTGTG
Optimized 3121	TCTGCTAATCTTGCTGCTACTAAAATGTCAGAGTGTGTACTTGGACAATCAAAAAGAGTT
Original 3121	GACTTCTGCGGCAAGGGATACCACCTGATGAGCTTCCCCCAGTCTGCTCCACACGGCGTC
Optimized 3181	GATTTTTGTGGAAAGGGCTATCATCTTATGTCCTTCCCTCAGTCAGCACCTCATGGTGTA
Original 3181	GTGTTCCTGCACGTCACCTACGTGCCTGCCCAGGAGAAGAACTTCACCACTGCCCCCGCT
Optimized 3241	GTCTTCTTGCATGTGACTTATGTCCCTGCACAAGAAAAGAACTTCACAACTGCTCCTGCC
Original 3241	ATCTGCCACGACGGCAAGGCTCACTTCCCAAGGGAAGGTGTCTTCGTGTCAAACGGCACC
Optimized 3301	ATTTGTCATGATGGAAAAGCACACTTTCCTCGTGAAGGTGTCTTTGTTTCAAATGGCACA
Original 3301	CACTGGTTCGTCACTCAGAGAAACTTCTACGAGCCTCAGATCATCACCACTGACAACACT
Optimized 3361	CACTGGTTTGTAACACAAAGGAATTTTTATGAACCACAAATCATTACTACAGACAACACA
Original 3361	TTCGTGTCCGGAAACTGCGACGTCGTGATCGGTATCGTCAACAACACCGTGTACGACCCA
Optimized 3421	TTTGTGTCTGGTAACTGTGATGTTGTAATAGGAATTGTCAACAACACAGTTTATGATCCT
Original 3421	CTGCAACCTGAGCTGGACAGCTTCAAGGAGGAACTGGACAAATACTTCAAGAACCACACC
Optimized 3481	TTGCAACCTGAATTAGACTCATTCAAGGAGGAGTTAGATAAATATTTTAAGAATCATACA
Original 3481	TCTCCCGACGTGGACCTGGGTGACATCAGCGGAATCAACGCTTCTGTCGTGAACATCCAG
Optimized 3541	TCACCAGATGTTGATTTAGGTGACATCTCTGGCATTAATGCTTCAGTTGTAAACATTCAA
Original 3541	AAGGAGATCGACCGTCTGAACGAAGTGGCTAAGAACCTGAACGAATCCCTGATCGACCTG
Optimized 3601	AAAGAAATTGACCGCCTCAATGAGGTTGCCAAGAATTTAAATGAATCTCTCATCGATCTC
Original 3601	CAAGAGCTGGGCAAGTACGAACAGTACATCAAGTGGCCTTGGTACATCTGGCTGGGTTTC
Optimized 3661	CAAGAACTTGGAAAGTATGAGCAGTATATAAAATGGCCATGGTACATTTGGCTAGGTTTT
Original 3661	ATCGCTGGCCTGATCGCCATCGTCATGGTGACCATCATGCTGTGCTGCATGACTAGCTGC
Optimized 3721	ATAGCTGGCTTGATTGCCATAGTAATGGTGACAATTATGCTTTGCTGTATGACCAGTTGC
Original 3721	TGCTCTTGCCTGAAGGGCTGCTGCTCATGCGGTTCCTGCTGCAAGTTCGATGAAGACGAT
Optimized 3781	TGTAGTTGTCTCAAGGGCTGTTGTTCTTGTGGATCCTGCTGCAAATTTGATGAAGACGAC
Original 3781	TCCGAGCCCGTTCTCAAAGGAGTGAAGTTGCATTACACATAA
	TCTGAGCCAGTGCTCAAAGGAGTCAAATTACATTACACATAA
S1 HA-TM-CT	CAGATCCTGGCTATCTACTCTACTGTGGCCTCCAGCCTGGTGCTGCTGGTCTCCCTGGGTGCTATCTCTTTCTGGATGTGCTCTAACGGCTCACTGCAGTGCCGCATCTGCATCTAA
S2 SP	ATGAAGTTCCTGGTGAACGTCGCCCTGGTGTTCATGGTGGTCTACATCTCCTACATCTACGCTGCCGCT

## Data Availability

Data supporting the findings of this study are contained within the article.
